# Continuous femoral nerve block is more effective than continuous adductor canal block for treating pain after total knee arthroplasty: A randomized, double-blind, controlled trial

**DOI:** 10.1097/MD.0000000000017358

**Published:** 2019-09-27

**Authors:** Michał Borys, Michał Domagała, Krzysztof Wencław, Joanna Jarczyńska-Domagała, Mirosław Czuczwar

**Affiliations:** a Second Department of Anesthesia and Intensive Care, Medical University of Lublin; b Faculty of Medicine and Health Sciences, Jan Kochanowski University, Kielce; c Department of Anesthesia and Intensive Care, St. Luke's Hospital; d Physiotherapy, Trauma, and Orthopedic Surgery Department, St. Luke's Hospital, Konskie, Poland.

**Keywords:** adductor canal block, femoral nerve block, patient-controlled analgesia, postoperative analgesia, total knee arthroplasty

## Abstract

**Objectives:**

Previous studies comparing adductor canal block (ACB) with femoral nerve block (FNB) are inconclusive with regard to patient-controlled analgesia (PCA) induced by opioids. Moreover, some postoperative pain severity results differ between previous randomized controlled trials (RCTs). The primary aim of the current study was to compare total intravenous morphine consumption administered via PCA during the first postoperative day in continuous FNB and ACB groups after total knee arthroplasty (TKA). Secondary aims included evaluation of postoperative pain via a visual analog scale, degree of knee extension, quadriceps muscle strength, and ability to sit, stand upright, and walk.

**Methods:**

The study was a RCT. Inclusion criteria were presence of gonarthrosis, age >18 and <75 years, and scheduled for TKA under single-shot spinal anesthesia.

**Results:**

A number of morphine uses was lower in the FNB group than in the ACB group (14, range 12–15 vs 20, range 18–22; *P* = .0001), and they perceived less severe pain at the 8th (*P* = .00003) and 24th hours. However, ACB was significantly superior with regard to most of the other parameters pertaining to mobility, including muscle strength at the 8th and 24th hours, degree of knee extension at the 8th hour, sitting at the 8th hour, standing upright at the 24th hour, and walking at the 24th and 48th hours.

**Discussion:**

FNB was associated with the perception of less severe pain after TKAs. However, ACB was associated with earlier mobility rehabilitation.

## Abstract 2

Supplemental Digital Content is available in the text

## 1 Introduction

Total knee arthroplasty (TKA) is one of the most common surgeries in developed countries.^[1–3]^ Several hundred thousand TKAs are performed every year in the United States.^[4]^ General and central neuraxial anesthesia (spinal and/or epidural) are anesthetic options for TKA.^[5]^ Neuraxial anesthesia may be advantageous over general anesthesia due to an associated reduction in the duration of the hospital stay, reduced postoperative pain, nausea and vomiting, and less cardiovascular and pulmonary complications.^[6,7]^ Epidural analgesia, the most commonly used continuous neuraxial method, may be extended to postoperative analgesia but it can also immobilize the opposite limb, delaying patient rehabilitation.^[8,9]^

Alternative regional techniques to extend pain treatment after TKA include femoral nerve block (FNB) and adductor canal block (ACB). These regional methods limit postoperative analgesia to the operated limb. ACB is theoretically advantageous over FNB due to blocking of the saphenous nerve (sensual) and the preservation of knee mobilization.^[10]^ However, postoperative pain can also reduce the effectiveness of the rehabilitation process.^[11,12]^ In most randomized controlled trials (RTCs) comparing ACB with FNB, no significant difference in pain intensity at rest has been reported.^[13–15]^ In 2 studies, however, lower pain intensity was detected in the ACB groups.^[16,17]^ Grevstad et al.^[16]^ reported ACB superiority at the 8th hour, and Zhang et al^[17]^ reported it at the 24th hour. Conversely, lower pain severity after FNB has only been reported in 1 RCT, at the 8th hour.^[14]^ In 3 separate studies comparing ACB and FNB with regard to morphine consumption administered by way of patient-controlled analgesia (PCA) pumps, there were no significant differences between the 2.^[13,14,18]^

The present study comparing the postoperative features of FNB and ACB after TKA was conducted due to inconclusive and conflicting findings in previous RCTs. The primary aim of the study was to evaluate postoperative pain intensity using intravenous morphine consumption administered via a PCA pump. Secondary aims included the evaluation of postoperative pain via a visual analog scale (VAS), and assessment of the rehabilitation process as indicated by degree of knee extension, quadriceps muscle strength, and ability to sit, stand upright, and walk.

## 2 Materials and methods

The study was a randomized, controlled, double-blind trial conducted in an orthopedic ward of a district hospital. Before patient recruitment, the study protocol was approved by the bioethics committee of the Medical University of Lublin, Lublin, Poland (permit number KE-0254/188/2016), and it was registered at ClinicalTrials.gov (NCT03143738). Written informed consent was obtained from every patient, and the study was conducted in accordance with the tenets of the *Declaration of Helsinki* for medical research involving human subjects.

The inclusion criteria included the presence of gonarthrosis, age >18 and <75 years, and scheduled for TKA under single-shot spinal anesthesia. Patients were excluded from the study if they had known coagulopathy or epilepsy, had depression or were receiving antidepressant drug treatment, had used any painkiller before the surgery, were addicted to alcohol or recreational drugs, or had any allergy to local anesthetics or other drugs used during the perioperative period. In every patient, before surgery knee flexibility and the ability to sit and walk were assessed by a physiotherapist.

### 2.1 Intervention

The patients were randomly allocated to 1 of 2 groups via computer-generated randomization conducted by a team member who was not involved in operations or patient assessment. The same team member prepared opaque envelopes in which the intervention type was concealed. These envelopes were opened a few minutes before attempting the regional block.

**Figure 1:**
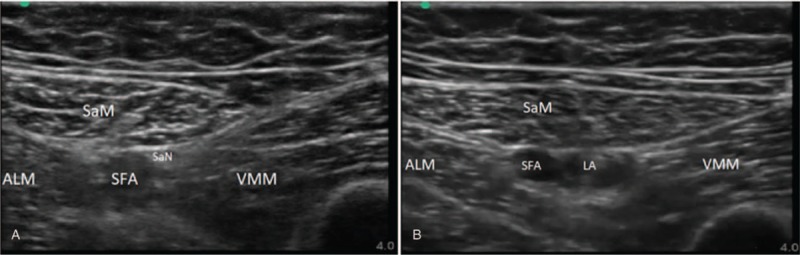
A. Ultrasound scanning before catheter implantation. B. Local anesthetic deposition in the area of saphenous nerve. ALM = adductor longus muscle, LA = local anesthetic, SaM = sartorius muscle, SaN = saphenous nerve, SFA = superficial femoral artery, VMM = vastus medialis muscle.

Patients were anesthetized prior to surgery by 1 of 2 physicians. Both anesthesiologists performed a subarachnoid block using a 27-gauge pencil-point needle with a solution of hyperbaric bupivacaine (0.5% Marcaine Heavy Spinal, Astra Zeneca, Dublin, Ireland). At the end of the surgery, the groin, distally to the inguinal ligament, or the adductor canal were scanned with a linear probe (15 MHz) to identify appropriate settings for the ultrasound apparatus (SonoSite M Turbo, Bothell, WA), based on the randomization. Using ultrasound guidance, a catheter dedicated to nerve blocks (Contiplex C, B.Braun, Melsungen, Germany) was placed next to the femoral nerve or the saphenous nerve (Fig. 1). To reduce the risk of catheter misplacement, the needle was inserted out of the plane of the ultrasound probe in order to place the catheter longitudinally to the nerve.

### 2.2 Pain treatment

At the end of the surgery, an elastomeric pump (Easypump B.Braun; 270 mL volume, 5 mL/hour flow rate) delivering 0.2% ropivacaine was connected to the catheter. Patients received intravenous morphine via a PCA pump (Medima S-PCA, Warsaw, Poland) with settings of bolus 1 mg and a lockout period of 10 minutes. Every patient received the non-opioid painkillers paracetamol (every 6 hours) and metamizole (every 6 hours). If postoperative pain exceeded 40 mm on a VAS scale, up to 2 extra doses of morphine (5 mg) were administered as rescue analgesia.

### 2.3 Patient assessment

At the scheduled time-points, a dedicated physiotherapist assessed pain severity at rest, operated knee mobility, ipsilateral quadriceps femoris muscle strength, and ability to sit, stand upright, and walk. The physiotherapist used the six-grade Lovett's scale to measure muscle strength, in which 0 denotes no muscle contractility and 5 denotes the complete range of motion against gravity, with full resistance. Patients were assessed at the 8th, 24th, and 48th postoperative hours, and just before they were discharged from the orthopedic ward.

### 2.4 Primary and secondary outcomes

The primary outcome in the study was the difference in the total consumption of intravenous morphine delivered via PCA pump during the first postoperative day between the ACB group and the FNB group. The secondary outcomes included pain intensity measured via a VAS, operated knee extension, ipsilateral quadriceps muscle strength, and ability to sit, stand upright, and walk. All secondary outcomes were measured by the same physiotherapist 4 times after the surgery.

### 2.5 Statistical analysis

Analysis of variance (ANOVA) and Student *t* test were used to analyze parametric data. Tukey's honestly significant difference test was used for post-hoc analysis. Results obtained using the VAS are presented as means and confidence intervals. Statistics derived from nonparametric data were calculated using the Mann–Whitney *U* test or the Kruskal–Wallis test by ranks, and are presented as medians and interquartile ranges. All measurements were performed using Statistica 13.1 software (Stat Soft. Inc., Tulsa, OK).

### 2.6 Power analysis

The sample size was calculated for the primary outcome, based on preliminary results. Before commencing the trial, we evaluated 2 groups of 10 patients; an FNB group and an ACB group. The mean numbers of uses of intravenous morphine were 13 in the FNB group and 20 in the ACB group. The computed number of individuals required in each group was 44 (power 0.9, alfa 0.05). We decided to recruit 100 patients (50 per group).

## 3 Results

The study was conducted from June 2017 to July 2018, and 85 patients completed the study (flow diagram). Patient demographics are shown in Table 1. Patient raw data are available as Supplemental Digital Content. There were no significant differences in any of the demographic parameters between the 2 groups. In 15 patients’ follow-up analysis was discontinued due to regional block failure or catheter displacement (8 in the FNB group and 7 in the ACB group).

**Table 1:**
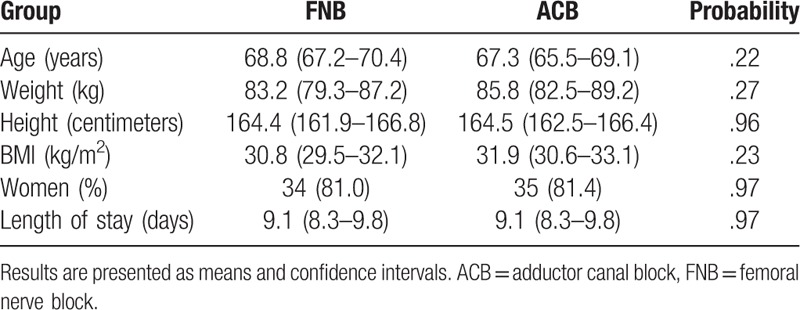
Patient demographics.

### 3.1 Primary outcome

There was a significant difference in the total number of morphine uses during the first postoperative day between the 2 groups (sum of ranks 1114 in the FNB group vs 2642 in the ACB group; *U* = 112, *P* = .0001). The number of uses per patient was lower in the FNB group (14, range 12–15) than it was in the ACB group (20, range 18–22).

### 3.2 Secondary outcomes

#### 3.2.1 Pain severity

**Figure 2:**
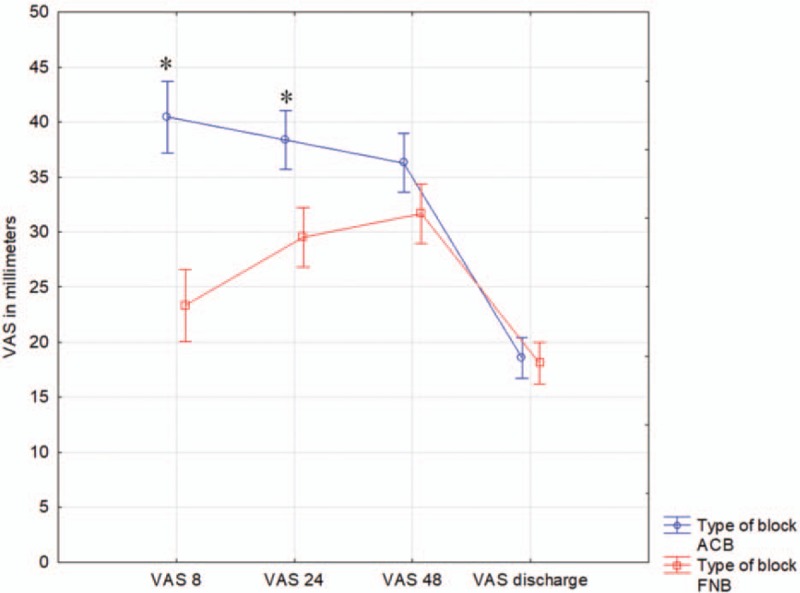
Pain severity presented on visual analog scale (VAS) in 2 groups of patients. Pain intensity was measured at 8th, 24th, 48th hours, and before patient discharge. Data are presented as means and confidence intervals. ∗ denotes probability below .05.

In 2 of 4 pain assessments performed at rest, patients in the FNB group perceived significantly lower pain than patients in the ACB group (Fig. 2). At the 8th hour, in the FNB group the mean pain severity measured via the VAS was 23 (range 20–27) and in the ACB group it was 40 (range 37–43) (*P* = .00003). The respective mean scores and ranges in the FNB and ACB groups at the subsequent time-points were 30 (27–32) vs 38 (36–41) at the 24th hour (*P* = .0001), 32 (29–34) vs 36 (34–39) at the 48th hour (*P* = .23), and 18 (16–20) vs 19 (17–20) at the time of discharge (*P* = 1.0).

#### 3.2.2 Quadriceps muscle strength

Quadriceps femoris muscle strength as measured by the physiotherapists via the Lovett's scale differed significantly in the FBA and ACB groups in 2 of 5 measurements. The data are presented in detail in Table 2, and are represented as medians and interquartile ranges. Probability was calculated using the Mann–Whitney *U* test.

**Table 2:**
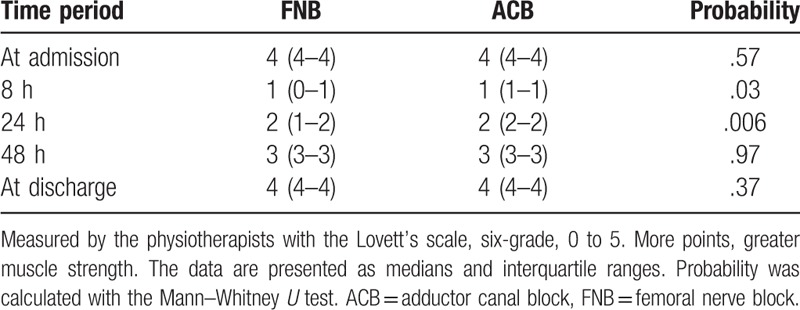
Quadriceps femoris muscle strength.

#### 3.2.3 Knee extension

Knee extension was assessed after a maximal, voluntary knee flexion. Thus, this parameter was correlated with quadriceps femoris muscle strength. At the 8th hour, patients in the ACB group could extend knee easier, 10 degrees (all patients) vs 0 degrees (range 0–10 degrees) (sum of ranks 2172 vs 1483, *U* = 580, *P* = .001). In contrast, at discharge knee flexion was more prominent in the FNB group, 100 degrees (range 90–100 degrees) vs 90 degrees (range 90–100 degrees) (sum of ranks 2046 vs 1609, *U* = 663, *P* = .02. There were no significant differences between the 2 groups at other time-points.

#### 3.2.4 Sitting, standing upright, and walking

At several time-points, there were significant differences in ability to sit, stand upright, and walk between the FNB group and the ACB group. More patients were able to sit at the 8th hour after continuous ACB (sum of ranks 1996 for ACB vs 1659 for FNB, *U* = 756, *P* = .007). More patients in the ACB group could stand upright at the 24th hour (sum of ranks 2365 for ACB vs 1290 for FNB, *U* = 387, *P* = .0001). More patients in the ACB group could walk at the 24th and 48th hours than in the FNB group. At the 24th hour the sum of ranks was 2059 for ACB vs 1596 for FNB (*U* = 693, *P* = .001), and at the 48th hour it was 2253 for ACB vs 1402 for FNB (*U* = 500, *P* = .001).

## 4 Discussion

The results of the present study demonstrate the superiority of continuous FNB in comparison to ACB with regard to the primary outcome, PCA-administered intravenous morphine. The total consumption of morphine was significantly lower in the FNB group than in the ACB group. This finding was consistent with pain severity measured using a VAS (Fig. 2). During the first postoperative day, patients in the FNB group perceived less severe pain than those in the ACB group.

Notably however, ACB was superior to FNB with regard to quadriceps femoris muscle strength, and because of that, patients in the ACB group were able to sit, stand, and walk after a shorter period. ACB patients were able to extend the operated knee easier at the 8th postoperative hour, but not at the time of discharge from the orthopedic ward.

Interestingly, none of the previously reported RCTs have demonstrated a significant difference in morphine consumption, including three studies in which PCA was utilized.^[13,14,18]^ Of these three studies, only Kim et al^[14]^ reported a significant difference in pain intensity—albeit only at a single time-point (8th hour)—in favor of FNB. In both studies in which pain severity was lower after ACB at some time-points,^[16,17]^ the PCA technique was not used as part of multimodal analgesia, and pain severity was assessed solely via a VAS. In some circumstances, as well as reflecting pain intensity, VAS scores can be influenced by associated factors as well.^[19]^ In contrast, PCA seems to be more accurate and better correlated with pain severity, which may be important during the process of establishing new techniques in clinical practice.^[20]^ In the present study, VAS results were consistent with morphine consumption. Patients in the FNB group perceived less severe pain than those in the ACB group.

Aside from pain intensity, most of our secondary outcomes were in favor of ACB. To our knowledge, quadriceps femoris muscle strength was measured in a similar manner (six-point grading system) in 2 previous studies.^[15,17]^ Zhang et al^[17]^ reported significantly stronger muscle strength at the 4th, 24th, and 48th postoperative hours in an ACB group. Findings were similar in the present study at the 8th and 24th hours, but not at the 48th hour (Table). A validated method of assessing patient mobilization ability, the Time Up and Go (TUG) test, has only been used in 2 reported studies.^[13,18]^ Of these, only Hegazy et al^[18]^ reported earlier mobilization ability in patients after ACB. In the current study, at several time-points the advantage of ACB with regard to ability to sit, stand upright, and walk was evident. However, in comparison with the TUG test the present findings are less precise.

The current study had some limitations. We did not implement a validated test to measure patient mobilization ability, and quadriceps muscle strength was only evaluated manually by a physiotherapist. Furthermore, we only measured pain intensity at rest. Lastly, we did not investigate differences in the length of patient stays in hospital. The reason for this is related to departmental policy pertaining to knee mobility. Patients in both groups were rehabilitated until the angle of knee extension measured was at least similar to that measured on admission.

In conclusion, in the current study FNB was superior to ACB with regard to intravenous morphine consumption after TKA. This observation was consistent with pain intensity measured via a VAS during the first postoperative day. However, quadriceps muscle strength, degree of knee extension, and ability to sit, stand upright, and walk were better in the ACB group. We believe that additional—particularly high-volume—studies are needed to facilitate a better understanding of the roles of ACB and FNB after TKA.

## Author contributions

**Conceptualization:** Michał Borys, Michał Domagała, Joanna Jarczyńska-Domagała, Mirosław Czuczwar.

**Data curation:** Michał Borys.

**Formal analysis:** Michał Borys.

**Investigation:** Michał Domagała, Krzysztof Wencław, Joanna Jarczyńska-Domagała, Mirosław Czuczwar.

**Methodology:** Michał Borys.

**Project administration:** Michał Borys, Michał Domagała, Krzysztof Wencław.

**Resources:** Michał Domagała, Joanna Jarczyńska-Domagała.

**Supervision:** Michał Borys.

**Validation:** Krzysztof Wencław.

**Writing – original draft:** Michał Borys.

**Writing – review & editing:** Michał Borys, Mirosław Czuczwar.

Michał Borys orcid: 0000-0002-6183-811X.

## Supplementary Material

Supplemental Digital Content
